# Aquaporins in Cancer Biology

**DOI:** 10.3389/fonc.2022.782829

**Published:** 2022-06-29

**Authors:** Chul So Moon, David Moon, Sung Koo Kang

**Affiliations:** ^1^ Department of Otolaryngology-Head and Neck Surgery, The Johns Hopkins Medical Institution, Baltimore, MD, United States; ^2^ HJM Cancer Research Foundation Corporation, Lutherville, MD, United States

**Keywords:** AQP in carcinogenesis, targeted therapy, genomic instability, hallmarks of cancer, AQP expression, water channel, glycerol channel

## Abstract

Aquaporins (AQPs) are a family of transmembrane water channel proteins, which were initially characterized as a novel protein family that plays a vital role in transcellular and transepithelial water movement. AQP1, AQP2, AQP4, AQP5, and AQP8 are primarily water selective, whereas AQP3, AQP7, AQP9, and AQP10 (called “aqua-glyceroporins”) also transport glycerol and other small solutes. Recently, multiple reports have suggested that AQPs have important roles in cancer cell growth, migration, invasion, and angiogenesis, each of which is important in human carcinogenesis. Here, we review recent data concerning the involvement of AQPs in tumor growth, angiogenesis, and metastasis and explore the expression profiles from various resected cancer samples to further dissect the underlying molecular mechanisms. Moreover, we discuss the potential role of AQPs during the development of genomic instability and performed modeling to describe the integration of binding between AQPs with various SH3 domain binning adaptor molecules. Throughout review and discussion of numerous reports, we have tried to provide key evidence that AQPs play key roles in tumor biology, which may provide a unique opportunity in designing a novel class of anti-tumor agents.

## Introduction

A group of aquaporins (AQPs) was discovered as a family of transmembrane water channel proteins, with each isotype playing a vital role in water homeostasis. AQPs are widely distributed in almost all tissues throughout biological systems. Since the discovery of the protein and genomic structure in the early 1990s (1, 2), AQPs have been shown to be key players in both the transcellular and transepithelial water movement (1–3). Biochemically, two regions of the channel are critical for AQP function, the hourglass model initially proposed by Agre et al. (1–6). The asparagine–proline–alanine sequences (NPA motifs) are highly conserved in AQP water channel family. Studies of AQP1 structure suggested that the two NPA motifs are in the narrow central constriction of the channel, serving to bind water molecules for selective and efficient water passage (1–6). Most AQPs are localized in the plasma membrane and several isoforms are found in cytoplasmic compartments, such as the endoplasmic reticulum (ER), where their delivery to the plasma membrane is regulated and vital in the modulation of water transfer (5, 6). Through this process, AQPs play crucial roles during various processes of cellular homeostasis.

AQPs are expressed in many epithelial, endothelial, and other tissues, with at least 13 AQPs and their genomic localizations being described in mammals (3–5). Initial studies have classified at least two groups of AQPs depending on their water transport and other transporter capabilities (3, 5, 6). AQP1, AQP2, AQP4, AQP5, and AQP8 function as water selective channels, with recent definitive evidence for other transporter capabilities, whereas AQP3, AQP7, AQP9, and AQP10 also transport glycerol and other small solutes in addition to their primary function as water channels (5, 6). Various follow-up studies have indicated that AQPs have other important roles in the transport of various other molecules, including carbon dioxide, metalloids, nitric oxide, ammonia, urea, and various ions (5, 6). Since the initial description of AQP1 induction during erythrogenesis (7), the genetic regulation of various AQPs has been studied. In addition, whereas AQPs can also be modified by phosphorylation of various amino acids, their gating activity may also be modified depending on the intracellular and extracellular environment, including pH, oxygen pressure, temperature, and solute gradient (5, 6, 8–10).

AQPs are historically known to be passive transporters of water. As recently reviewed by Kitchen et al., evidence in the last decade has revealed diverse functions of AQPs beyond water homeostasis (11). Lessons learned from AQP knockout studies in whole animals and cultured cells, in addition to naturally occurring human mutations, suggest that the transport of neutral solutes through AQPs has important physiological roles. Moreover, AQPs are proposed to play a crucial role during cell signaling for volume regulation and in controlling the subcellular localization of other proteins through the formation of macromolecular complexes. Most recently, new roles of AQPs have been characterized in terms of cellular proliferation and survival. For example, each type of AQP can participate as an important player during human carcinogenesis by facilitating proliferation, cellular migration with accompanying invasion, and metastasis in addition to drug resistance and potential prognostic markers in specific cancer type(s) (10–13). Recently, with the discovery of superaquaporin AQP11 (14) and AQP12 (15), the role of AQPs in cellular homeostasis has begun to be elucidated. Of note, whereas most AQPs are located in the plasma membrane to drive osmotic gradient-dependent water transport, AQP11 and AQP12, as super-aquaporins, are expressed in the cytoplasm to regulate intracellular water transport, organelle volume, or intra-vesicular homeostasis (16). AQP11 is mainly localized in the ER and is permeable to water, glycerol, and H2O2 (14, 17). Moreover, AQP11 expression is correlated with a favorable overall survival among patients with ovarian cancer (18).

The initiation and progression of cancer is promoted by genetic and epigenetic changes in cellular DNA, which renders cells to succumb to excessive proliferation accompanied by certain escape mechanisms that are designed for survival and migration. Signaling pathways that control cell growth and division, cell death, cell fate, and cell motility are drastically altered during human carcinogenesis, which ultimately become the driving force for cancer progression. Importantly, in carcinogenesis, certain types of mutations in proto-oncogenes lead to the activation of oncogenes and other types of mutations, resulting in compromised function of tumor suppressors. So far, numerous studies have demonstrated important roles of several key kinases, including phosphoinositide 3-kinase (PI3K)–Akt–driven and Ras-ERK–driven pathways in tumor cell proliferation. Likewise, cellular growth is partially controlled by unique signaling from the extracellular to intracellular space. Since this was first demonstrated by EGF to EGFR-mediated signaling pathways (19, 20), we now understand that similar signaling processes play a vital role in other key processes, such as migration of tumor cells and cancer metastasis (21–23). On the basis of careful analysis of these kinase pathways, several new biological therapeutics have been designed and have shown clinical efficacy in the control of various types of solid tumor (23, 24), and some of these biological therapeutics target the crucial role of endothelial cells during tumor angiogenesis (25). The initial report from our group described the role of AQPs during colorectal development (26), and numerous follow-up studies have demonstrated the expression of AQPs in various resected cancer samples and *in vivo* rodent models (27). Moreover, prognostic implications of AQP expression in the lung, brain tumors, and human hemangioblastoma have been described, and the potential role of AQPs targeting has been discussed (28–32).

In this review, we outline the expression of AQPs in human and rodent models and discuss their potential role in modifying known biological pathways during human carcinogenesis. Although available data are in the early stage, we expect that the understanding of AQPs in tumor biology will broaden the knowledge of the interactions between tumor cells and their microenvironment. Of note, although we discuss the expected role of AQPs in cancer development and progression, we do not review AQP targeting therapeutics in detail, while we briefly discuss about a summary of overall development of AQP targeting therapeutics.

## Role of AQPs in Human Carcinogenesis

### Aquaporin 1

**Figure 1:**
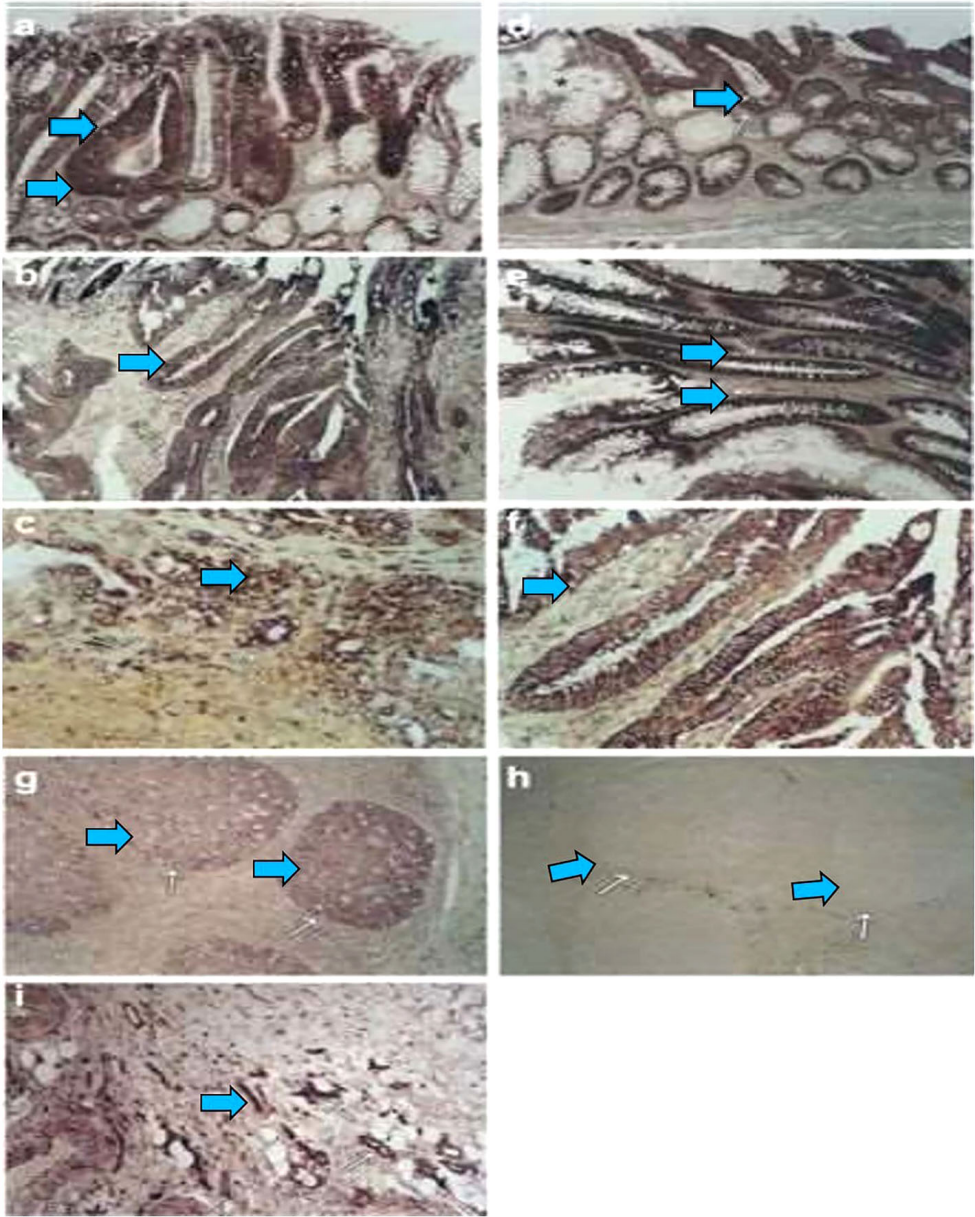
During colorectal carcinogenesis, expression of AQP1 and AQP5 is induced, which is examined by *in situ* hybridization. To confirm these findings *in vivo* and to evaluate the pattern of AQP induction during carcinogenesis, *in situ* hybridization with AQP1, AQP3, and AQP5 riboprobes generated by cloning the RT–PCR products, which was used to study the expression of these AQPs in colon cancer and in different stages of preneoplastic lesions. From five patients with colon cancer, 16 tissue samples were studied, including one patient with metastasis to the liver. Of these samples, 12 consisted of adenocarcinomas and the surrounding normal colonic tissue, whereas four were metastatic lesions from the liver. Of the five patients, staged surgically resected series were available for three patients. Expression of The AQP1 detected from staining with antisense riboprobe was identified colonic adenoma (**A**, arrow), primary colon cancer (**B**, arrow), and metastatic lesions in the liver (**C**, arrow). Likewise, the AQP5 expression is also detected in early adenoma with moderately dysplastic cells (**D**, arrow), late adenoma with severe dysplastic cells (**E**, arrow), and adenocarcinoma (**F**, arrow). There is almost no expression in the surrounding normal (**A**, **F**, star). As positive controls, the germinal centers of the tonsils were stained with antisense riboprobes of AQP5 **(G)**. Sense riboprobes of AQP1 (data not shown) and AQP5(h) is shown as negative control **(H)**. As an internal control, expression of AQP1 in the vascular endothelium is shown **(I)**. Adapted from (26).

An initial observation from our group following careful analysis of resected tumor samples indicated that co-expression of several AQPs contributes to human carcinogenesis, providing the first insight that AQPs are involved during various stages of carcinogenesis (26). As we discuss below, the fact that genomic instability is now an accepted theory of tumorigenesis (23) and the possible implications of human AQP expression as a driver of cancer *via* genomic instability suggests a link between AQP expression and genomic instability. mRNA expression of AQP1, AQP3, and AQP5 was examined in seven colon and colorectal cancer cell lines, and protein expression was confirmed in four of these cell lines. During colorectal carcinogenesis, mRNA expression of AQP1 and AQP5 was induced in early-stage disease (early dysplasia), with expression remaining stable through the late stages of colorectal carcinogenesis (**Figure 1**). Moreover, expression of AQP1 and AQP5 was demonstrated among metastatic spots in the liver. These findings demonstrated, for the first time, that the expression of several AQPs can be detected in tumor cells and is associated with early cancer development during colorectal cancer carcinogenesis. Several follow-up reports have confirmed the expression of AQP1 among various human malignancies including brain tumors, hemangioblastomas, multiple myeloma, lung cancer, and breast cancer, expanding the expression of AQPs to multiple cancer types (12, 31–33).

Studies by our group have demonstrated that AQP1 can induce NHI-3T3 cell proliferation and anchorage-independent growth (28) and that the promotion of anti-apoptosis seemed to be important underlying mechanism. As AQPs represent a family of transmembrane water channel proteins that are widely distributed in various tissues throughout the body and play a major role in transcellular and transepithelial water movement, their role in high vascular permeability and high interstitial fluid pressure among tissues and their role in the transport pathways for water within tumors have been extensively studied. Several studies suggested a crucial role of AQP1 and AQP4 in the vascular permeability of edematous brain tumors (29, 31). For example, although there was little AQP1 immunoreactivity in normal brain parenchyma, among astrocytomas, AQP1 was expressed in microvessel endothelia and neoplastic astrocytes. In metastatic carcinomas, AQP1 was present in microvessel endothelia and reactive astrocytes. AQP1 may participate in the formation of brain tumor oedema (31). AQP1 was found to be expressed in endothelial cells of non-fenestrated capillaries (34), human arteries (35), descending vasa recta kidney (36, 37), and peritoneal capillaries (38). Importantly, robust expression of AQP1 was detected among proliferating tumor microvessels in resected cancer samples (33). Report from Vacca et al. has demonstrated that, among plasma cell tumors, AQP1 expression is preferentially associated with microvessels of multiple myeloma and that the highest degree of expression occurs in active MM in parallel with enhanced angiogenesis, where AQP1 expression was more abundant among more immature neovessels than mature counterparts. This report further suggested an exciting possibility that AQP1 may promote angiogenesis, resulting in MM progression and become one of the first reports proposing a potential therapeutic vascular targeting of AQP1. The *in vivo* rat model and the chick embryo chorio-allantoic membrane (39, 40) also shed insight for the role of AQP1 during angiogenesis. Studies by Endo et al. have used two mammary carcinomas cell lines and three glioblastomas transplanted into rat and demonstrated that AQP1 water channel is heterogeneously expressed in tumor cells and their vasculature and that the level of expression is determined not only by the specific cellular origin of the tumor but also by the location of the tumor in the host. A follow-up study provided more direct insights into the role of AQP1 during blood vessel formation. In wild-type and AQP1-null model mice, both of which were implanted with melanoma cells (41), there were remarkably impaired tumor growth in AQP1-null mice, after subcutaneous or intracranial tumor cell implantation, which were accompanied by reduced tumor vascularity and extensive necrosis. Mechanistically, whereas adhesion and proliferation were similar in primary cultures of aortic endothelia from wild-type and from AQP1-null mice, cell migration was greatly impaired in AQP1-deficient cells, with abnormal vessel formation *in vitro*. Stable transfection of non-endothelial cells with AQP1 or with a structurally different water-selective transporter (AQP4) accelerated cell migration and wound healing *in vitro*. Motile AQP1-expressing cells had prominent membrane ruffles at the leading edge with polarization of AQP1 protein to lamellipodia, where rapid water fluxes occur. Our findings support the fundamental role of water channels in cell migration, which is central to diverse biological phenomena including angiogenesis, wound healing, tumor spread, and organ regeneration. Moreover, the same group confirmed that tumor cell migration and metastasis were increased by AQP1 expression, whereas inhibition of AQP1 expression resulted in a significant reduction in the growth of new blood vessels in the commonly used chorioallantoic membrane (CAM) model (42).

Hoque et al. initially reported the expression of AQP1 in resected lung cancer samples (28). A follow-up study by Yang et al. (43, 44) reported a positive relationship between the degree of AQP1 and AQP5 expression and intratumoral microvessel density, whereas in epithelial ovarian tumors, a similar relationship existed between the expression of AQP1 and AQP5 and ascites formation. Pan et al. (45) then demonstrated a positive correlation between the expression of AQP1 and the degree of vascular endothelial growth factor (VEGF) expression, implicating AQP1 in the progression of endometrial adenocarcinoma. Of note, AQP1 is expressed in brain tumors, which show similar findings to those of other tumors (31) and warrants more detailed discussion. In sections of normal brain, AQP1 expression was observed in a few (< 33%) microvessels. Among metastatic carcinomas, AQP1 expression was not detected among cancer cells and was detected in glioblastoma cells and microvascular endothelial cells, but not in normal brain parenchyma or normal microvessel endothelium.

Kaneko et al. (46), using the retinal vascular system, demonstrated hypoxia-induced AQP1 expression. Hypoxia is thought to be a common precursor to neovascularization in many retinal diseases, including diabetic retinopathy, and therefore, the expression pattern and function of AQP1 in human retinal vascular endothelial cells cultured under hypoxic conditions were examined. The levels of AQP1 mRNA and protein expression significantly increased under hypoxia, and of note, inhibition of VEGF signaling did not affect AQP1 expression. Reduction of AQP1 expression using siRNA and inhibition of VEGF signaling both significantly inhibited tube formation, and these effects were additive. These findings overall suggest that AQP1 is involved in hypoxia-inducible angiogenesis in retinal vascular endothelial cells through a mechanism that is independent of the VEGF signaling pathway.

Jiang et al. (47) demonstrated the expression of AQP1 among several cancer cell lines and in a human HT20 colon cancer cell model and, by adenovirus-mediated expression of AQP1, further showed that AQP1 could increase cellular membrane water permeability. These transfected cells demonstrated an enhanced cell migration rate in a wound healing assay and an invasive Transwell migration assay.

The expression of AQP1, AQP3, and AQP5 has been found to be increased among resected breast cancer samples and carries a prognostic importance (48, 49). AQP1 enhances tumor cell angiogenesis by signaling downstream of estrogen receptors, while, at the same time, estrogen can increase the expression of AQP1 through transcriptional regulation of the AQP1 promoter (48, 50). Among patients with HIF1-positive and HIF1-negative breast cancer, the expression of AQP1 was found to be positively correlated with HIF expression (51–53). In the report by Kaneko et al., human retinal vascular endothelial cellshuman retinal vascular endothelial cells (HRVECs) were cultured under normoxia or hypoxia to elucidate the mechanism of hypoxic induction of AQP1. The DNA sequence located from −1,338 to −1,334 bp is identical to the consensus sequence of the hypoxia-inducible factor 1 (HIF-1) binding sit, which seem to be responsible for hypoxia-induced AQP1 expression, whereas the chromatin immunoprecipitation assay showed that HIF-1α bound to the putative HIF-1 binding site. This report concluded that hypoxia-induced expression of AQP1 requires transcriptional activation, and the HIF-1 binding site of the 5′-promoter is necessary for transcriptional activation.

Of note, Esteva-Font s et al. investigated the consequences of AQP1 deficiency in mice that spontaneously develop well-differentiated, luminal-type breast adenomas with lung metastases [mouse mammary tumor virus-driven polyoma virus middle T oncogene (MMTV-PyVT)]. AQP1(+/+) MMTV-PyVT mice developed large breast tumors with total tumor mass tumor masses, which were greatly reduced in AQP1(−/−) MMTV-PyVT mice. CD31 immunofluorescence showed abnormal microvascular anatomy in tumors of AQP1(−/−) MMTV-PyVT mice, with reduced vessel density. Of note, HIF-1α expression was increased in tumors in AQP1(−/−) MMTV-PyVT mice. The number of lung metastases was significantly reduced among AQP1(+/+) MMTV-PyVT mice. These results overall suggest AQP1 as a key player during tumor angiogenesis and can be a potential drug target for solid tumors. It seems that the expression of AQP1 and HIF1 can augment each other, and this relationship may provide an important biological rationale of designing novel anti-AQP1 therapeutics (52, 53). In another study using various cancer cell lines transplanted into mice, tumor growth and progression were hampered in AQP1 KO mice, similar to prior reports (41, 42, 54, 55). Moreover, phosphorylation of p38 mitogen-activated protein kinase (MAPK) was induced by hypoxia, and specific inhibitors of p38 MAPK could concentration-dependently block those effects of hypoxia on AQP1 expression, whereas intracellular calcium ion [Ca(2+)] and protein kinase C (PKC) were shown to be responsible for the activation of p38 MAPK pathways (54).

Cellular migration involves five interdependent stages, polarization, protrusion, cell–matrix adhesion, and degradation of the extracellular matrix (ECM). Although it is not completely characterized, different AQPs play crucial roles in some or all of these steps. The impact of AQPs on each cell migration step can be modified by modulating the expression of key molecules, such as CDC42 or RAC and various ion transporters, and through changes in the cytoskeleton *via* actin or myosin. As an initial step of cell migration, polarization occurs *via* molecular machinery to generate various specialized structures in both the cell membrane and cytoplasm. Such structures involve various structure-specific events, including asymmetric distributions of organelles, domain-specific signaling, and different expression and distribution of various membrane channel proteins and signaling receptors (56, 57), which promote the synthesis of leading and trailing edges. These asymmetric domains in the membrane and cytoplasm seem to be regulated by the GTPase-like CDC42 [57.58], which can modulate the actin polymerization machinery (58, 59) through selectively recruiting partitioning defective proteins and atypical PKC. In the report by Hara-Chikuma et al., cellular migration was compared in primary cultures of proximal tubule cells from wild-type and AQP1 null mice (60). Migration of AQP1-deficient cells was reduced by >50% compared with wild-type cells, in the presence of a chemotactic stimulus. Comparable slowing of migration of AQP1-deficient cells was also found in an *in vitro* scratch assay of wound healing, with reduced appearance of lamella-like membrane protrusions at the cell leading edge. Expression of AQP1 in the AQP1-deficient cells corrected their migration defect. From *in vivo* model of acute tubular injury caused by 30 min of renal artery occlusion, at 3 to 5 days after ischemia-reperfusion, kidneys in AQP1 null mice demonstrated markedly increased tubular injury and cellular actin disorganization than kidneys in wild-type mice. These results provide evidence for the involvement of AQP1 in migration of proximal tubule cells and its protective role from proximal tubule to injury. As discussed above, understanding filopodial formation in motile cells is a pertinent task in cell biology (56–58). Of note, the report by Loitto et al. has demonstrated that expression of the human water channel AQP9 in different cell lines can induce the formation of numerous filopodial extensions (61). Further experiments have demonstrated a potentially important role of AQPs functioning both as a signaling participant during cellular migration. The number of filopodia is decreased by site-directed serine substitutions in putative PKC-binding or phosphorylation sites at amino acid positions 11 and 222 in AQP9. Importantly, the filopodial phenotype obtained with wild-type AQP9 is associated with elevated levels of active Cdc42, whereas serine-deleted mutants have reduced levels of GTP-Cdc42. Co-transfection with inhibitory N-WASP CRIB completely abolishes wild-type AQP9-induced filopodia formation. Active PKC(zeta) phosphorylates wild-type AQP9, and myristoylated PKC(zeta) pseudosubstrate inhibits the formation of filopodia in AQP9-expressing cells. Expression of wild-type AQP9, but not mock or serine substituted mutants, increases sensitivity to hypo-osmolaric conditions, yielding a rapid morphological rounding of cells and cell death starting as early as 24 h post-transfection. Overall, these findings suggest that the increased water influx through AQP9 is critically involved in the formation of membrane protrusions and that AQP9-induced actin polymerization is augmented by activation of Cdc42 and PKC(zeta). So far, at least four AQPs, including AQP1, AQP14, AQP15, and AQP19, are shown to be localized at the leading edges of migrating cells. Expression of these AQPs has been shown to be distributed together with three ion transporters, including the Na+/H+ exchanger, the Cl−/HCO3 exchanger, and the Na+/−HCO3 co-transporter. It was further proposed that synergistic action of these four AQPs and these three ion transporters may promote fluid influx and efflux (59– 62). A proposed mechanism by which AQPs may facilitate cell migration, which takes into consideration AQP polarization, is proposed. According to this hypothesis, actin de-polymerization and ion influx increase cytoplasmic osmolality at the front end of the migrating cell. Changes in actin polymerization and transmembrane ion movements (mediated by the ion exchangers) at the front end of migrating cells are well-documented. The Na+/H+ ion exchanger polarizes to the leading edge of migrating cells. It has been suggested that ion transporters mediate changes in cell volume during migration such that water enters through the plasma membrane at the leading end, producing localized cell swelling, and exits the plasma membrane from the rear, producing localized cell shrinkage. Consistent with the idea that water flows into and out of migrating cells are reports that migration can be inhibited or accelerated by changing the osmolality of the extracellular medium. Moreover, it is considered that, through various interactions between AQPs and other partner proteins, AQPs can promote various stages of cellular movements (63). In the report by Stroka et al., underlying mechanism regarding cell migration through physically confined spaces, even when typical hallmarks of 2D planar migration such as actin polymerization and myosin II-mediated contractility are inhibited. This report proposed a “Osmotic Engine Model” and demonstrate that directed water permeation is a major mechanism of cell migration in confined microenvironments. Using microfluidic and imaging techniques along with mathematical modeling, it has demonstrated that tumor cells confined in a narrow channel establish a polarized distribution of Na+/H+ pumps and AQPs in the cell membrane, which creates a net inflow of water and ions at the cell leading edge and a net outflow of water and ions at the trailing edge, leading to net cell displacement. Importantly, this report presents an alternate mechanism of cell migration in confinement that depends on cell-volume regulation *via* water permeation (63).

During migration, contact with the cellular matrix and basement membrane are essential to facilitate the extension, and, importantly, it has been reported that a compromised anchoring process can cause cellular protrusions to collapse (64). Cellular protrusions must attach to the ECM through integrin receptors, which then can initiate the formation of intracellular actin filaments (64, 65). Binding of the extracellular integrin receptors to the ECM can initiate a series of events, including clustering of integrins, with activation of various protein tyrosine kinases, including focal adhesion kinase (FAK) and associated small GTPases (64). Proper actin cytoskeleton structure and cell polarity are essential for precise positioning of focal adhesions for directional cell movement (65–67). Recently, interactions with key adhesion molecules have been described among several AQPs (AQP1–4), and it has been postulated that AQPs may influence the adhesive properties of cellular behavior.

Expression of AQP1 in mesenchymal stem cells can modulate the migration of stem cells (67–69). Meng et al. demonstrated that overexpression of Aqp1 promoted mesenchymal stem cell (MSC) migration, whereas depletion of Aqp1 impaired MSC migration in a rat model with a femoral fracture (69). When the green fluorescent protein (GFP)–labeled Aqp1 overexpressing MSCs were systemically injected into rats with a femoral fracture, there were significantly more GFP-MSCs found at the fracture gap in the Aqp1-GFP-MSC–treated group compared with the GFP-MSC group. Screening studies for several migration-related regulators have demonstrated that β-catenin and FAK were upregulated in the Aqp1-MSCs and downregulated in the Aqp1-depleted MSCs, whereas C-X-C chemokine receptor type 4 had no change. Furthermore, β-catenin and FAK were co-immunoprecipitated with Aqp1, and depletion of FAK abolished the Aqp1 effects on MSC migration. This study demonstrates that Aqp1 enhances MSC migration ability mainly through the FAK pathway and partially through the β-catenin pathway. Our finding suggests a novel function of Aqp1 in governing MSC migration, and this may aid MSC therapeutic applications. Of note, through various *in vitro* brining studies, the report further provided potential molecular mechanism for AQP1-mediated activation of the FAK and β-catenin pathway, which may result in integrin-mediated ECM attachment.

AQPs at the leading edges of migrating cells contribute to cell volume changes with energy efficient water transport and cytoskeletal modifications (55, 67–72). AQP1 overexpression in B16F10 melanoma cells and 4T1 mammary gland tumor cells facilitates cell migration and formation of lamellipodia by increasing membrane osmotic water permeability (27, 47, 70), allowing sufficient space for actin polymerization. Of note, AQP1 is also thought to be an ion channel, which is proposed to allow gated conduction of monovalent cations through the central tetrameric pore; this additional transporter activity from AQP1 may be important for colon cancer cell migration (73–77). Moreover, in samples from patients with cholangiocarcinoma, high AQP1 expression has been found to be consistent with reduced metastasis (78). Expression and transfection studies of AQPs in several commonly used human glioma cell lines suggested that AQP4 enhanced cell adhesion, which was abolished without AQP1. Monzani et al. (55) demonstrated that AQP1 knockdown dramatically impeded actin cytoskeletal organization in some cell line models. This phenomenon is based on the interaction between AQP1 and Lin-7/b-catenin, both of which play an important role during proper structural organization of filamentous actin (F-actin), suggesting that AQP1 functions as a key scaffolding protein. Furthermore, Jiang et al. (47) demonstrated that the suppression of AQP1 expression resulted in re-localization of actin in migrating cells and suppressed the activity of RhoA and Rac, which play crucial roles in actin filament regulation. One proposed mechanism involves the PDZ domain from Lin-7, through which rhotekin protein can bind to Lin-7 to inhibit Rho GTPase signaling. The interaction between Rhotekin and AQP1 can prevent binding between Lin-7 and Rhotekin, which, in turn, activates Rho GTPase to promote cell migration with proper cytoskeletal reorganization (47, 79, 80). In summary, it is now hypothesized that because of actin polymerization/depolymerization based on various AQP binding partners and transmembrane ionic fluxes mediated by AQPs adjacent to the leading edge of migrating cells, AQPs could thus facilitate cell protrusions that form during migration. It is important to note that AQP-dependent cell migration has potentially broad implications in angiogenesis, tumor metastasis, wound healing, glial scarring, and other events requiring rapid, directed cell movement. AQP inhibitors targeting these events, such as new agents, leading into tumor growth suppression and blockage of cancer invasion, can be designed on the basis of better mechanistic understanding of AQPs during cell migration.

Epithelial-to-Mesenchymal Transition (EMT) during cancer cell invasion and metastasis (80) is characterized by a series of processes, during which polarized epithelial cells go through biochemical and biological changes to obtain to the of mesenchymal cells. This is accompanied by a loss of cell polarity and reduced cell–cell attachment, which result in augmented invasive potential (81, 82). Epithelial cadherin (E-cadherin) is a transmembrane protein, which enables a tight connection between certain types of epithelial cells to surrounding cytoskeletal structures. This connection enables epithelial cells to maintain their differentiated morphology, which is different from that of mesenchymal cells (83, 84). Therefore, reduced function or expression of E-cadherin is an essential part of the EMT process and plays a key role in mammalian carcinogenesis (83, 84). A series of signaling cascades and certain growth factors are known to trigger EMT during carcinogenesis. Four of the most well characterized signaling molecules include epidermal growth factor (EGF), platelet-derived growth factor (PDGF), hepatocyte-derived growth factor, and transforming growth factor beta (TGF-β) (81–86). These growth factors are produced during inflammation, infection, and response to carcinogen exposures and are often directly released by tumor-associated stroma and tumor cells themselves (81).

In the last two decades, several defined transcription regulators (SNAI1, SNAI2, and zinc finger E-box binding homeobox 1) and SMADs and Twist have been extensively characterized. Although these transcriptional factors play both common and different roles in modulating cellular behavior in response to internal or external stresses, all have been shown to suppress the expression of E-cadherin (81, 87). So far, four AQPs (AQP1, AQP4, AQP5, and AQP9) have been linked to EMT in several cancer cell models. Moreover, in lung adenocarcinoma cells, AQP1 overexpression showed certain cellular characteristics of EMT, including decreased expression of E-cadherin and increased expression of known EMT regulated proteins, such as vimentin and N-cadherin (88, 89). The term EMT refers to a complex molecular and cellular process by which epithelial cells shed certain characteristics (such as cell–cell adhesion, planar and apical-basal polarity, and lack of motility) and acquire mesenchymal features (motility, invasiveness, and resistance to apoptosis) and the fact that AQP1 plays a crucial role during this process provides an important clue for the role of AQP1 during mammalian carcinogenesis.

### Aquaporin 3

**Figure 2:**
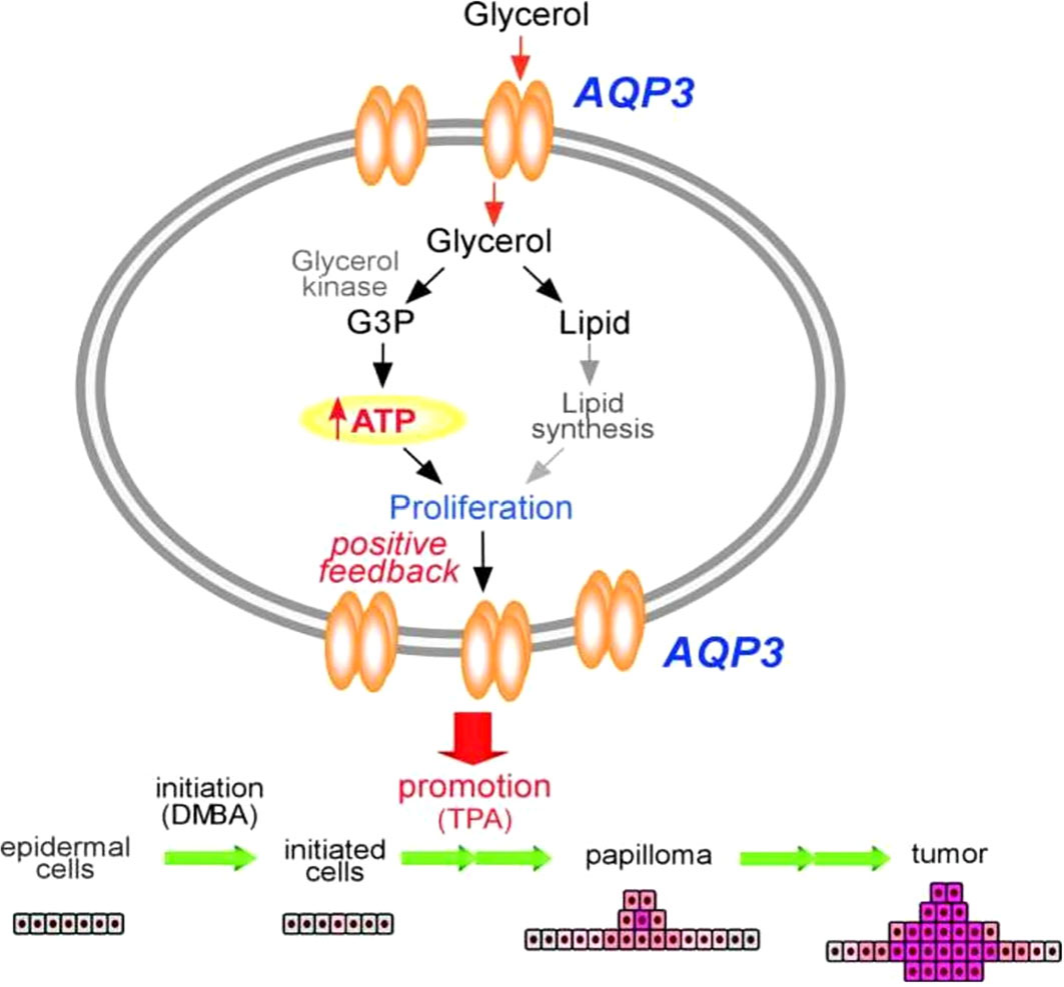
Mechanism of tumor promotion by AQP3 is demonstrated by studying AQP3 KO cell models for its proliferation assay from skin tumorigenesis. ATP production by AQP3-mediated glycerol transport may play an important role in the transformation of normal skin epithelial cells and growth of skin cancers. Modified from Reference 91. G3P, glycerol 3-phosphate; DMBA, 1,3-dimethylbutylamine; TPA, 12-O-tetradecanoylphorbol-13-acetate.

Unlike other AQPs, AQP3 is robustly expressed among basal epidermal cells in human skin. Of note, AQP3 expression is strongly increased in lung squamous cell carcinoma, but not in adenocarcinomas (89–91). However, the molecular mechanistic role underlying this strong expression of AQP3 in tumorigenesis, particularly in cellular metabolism, is poorly understood. Pioneering work using an AQP3 null mice model has provided initial insight. In this study, mice lacking AQP3 were resistant to the development of skin tumors after exposure to a common tumor initiator and chemicals such as phorbol ester (**Figure 2**) (90, 91). Interestingly, after exposure to a skin tumor initiator, a similar pattern of apoptotic responses was observed in both wild-type and AQP3-null mice. However, enhanced cell proliferation by phorbol ester was significantly reduced in the epidermis of AQP3-null mice. Reductions in epidermal cell glycerol, its metabolite glycerol-3-phosphate, and ATP were observed in AQP3 deficient mice; these changes were related to the role of AQP3 as a glycerol transporter, which was confirmed by the fact that additional glycerol corrected the suppressed cell proliferation and reduced the cellular ATP content. The role of AQPs in cellular proliferation predicted AQP-based energy metabolism, providing the first evidence for the role of AQPs in energy production, In **Figure 2**, the potential pathways regarding “how AQP3-mediated glycerol passage of transport-dependent ATP leads to transformation of normal skin epithelial cells and growth of skin. The principal finding from this report is the remarkable resistance of AQP3-deficient mice to the development of skin tumors. Impaired phorbol ester 12-O-tetradecanoylphorbol 13-acetate (TPA)-mediated proliferation was seen in the AQP3-deficient epidermis during the tumor promotion step, and the author postulated that this is due to the lack of promotion of the initiated cells, resulting in the absence of papillomas (91). Additional reports using colorectal, lung, and prostate cell lines and tissue samples also predicted the role of AQP3 in human carcinogenesis (92–94).

Human EGF (hEGF) can upregulate the expression of AQP3 protein and the migration ability of the human colorectal carcinoma cell line HCT116 in a dose- and time-dependent manner, for which AQP3 expression is partially responsible (93). Of note, the enhanced migration ability of HCT116 cells was blocked by the AQP3 inhibitor, CuSO4. Overexpression of AQP3 induced by hEGF was inhibited by a PI3K/AKT inhibitor, but the ERK inhibitor had a minor effect on the hEGF-induced AQP3 upregulation. Moreover, the expression of AQP3 in resected colorectal cancer samples was associated with lymph node and distant metastasis. In the case of lung cancer (93), although AQP3 is expressed in the normal respiratory tract and plays an important role in the maintenance of water homeostasis, similar to AQP1, expression of AQP3 also participates in pulmonary carcinomatosis (89). In the case of prostate cancer, based on a small number of samples, the report by Ismael et al. has demonstrated that inhibition of AQP3 increases the sensitivity of prostate cancer cells to cryotherapy (94). Cryotherapy is an effective therapy for localized or locally advanced prostate cancer, and a complete ablation of cancer tissue sometimes fails and results in disease recurrence. Therefore, a second synergistic therapy is of great potential benefit. Some synergistic cell killing has been achieved with concomitant cryotherapy and chemotherapy, radiotherapy, hyperthermia, and apoptosis inducing ligands; therefore, the report by Ismael et al. proposed “inhibition of AQPs” as a further therapeutic target that may be synergistic with cryotherapy.

Finally, a large-scale expression study based on using different types of tumor samples (90) demonstrated significant AQP3 protein expression in resected samples from pituitary adenomas, salivary gland tumors, thymic tumors, germinal cell tumors of the ovary and testis, urothelial carcinoma of the bladder, adenocarcinoma of the lung and prostate, squamous cell carcinomas of the skin, esophageal cancer, cancer of the uterine cervix, and apocrine carcinoma of the breast.

The suppression of AQP3 expression among human esophageal and oral squamous cell carcinoma cells resulted in reduced phosphorylation of FAK, with reduced adhesion and increased cell death (95). Cancer cells metastasize through ameboid and mesenchymal-dependent movement or through cell sheets, strands, and clusters, which are components of the collective migration processes (95–97). Invadopodium can be released from the leading edge of filopodia, which is firmly based on collagen fibers of the ECM (97, 98). Zinc-dependent matrix metalloproteinases (MMP) and other types of serine proteases then break ECM fibers (99, 100) and play key roles in the proper action of the invadopodium. Of note, at least four AQPs (AQP1, AQP3, AQP4, and AQP9) have been shown to modulate the activity of specific MMPs, which appears to be a key mechanism underlying the promotion of cellular local invasion and cancer cell metastasis by AQPs. AQP1 expression in lung cancer cells has been shown to be related to the expression of MMP2 and MMP9 (101). Likewise, AQP3 expression in gastric cell lines was correlated with MMP2, MMP9, and membrane type 1 (MT1)–MMP expression, which mediate upregulation of PI3K signaling (102). In some melanoma cancer cells, PI3K activity can modulate a unique interaction of MT1-MMP with MMP-2. PI3K enhances enzymatic activity from MT1-MMP, resulting in rapid activation of pro-MMP into its fully active enzyme conformation. Active MT1-MMP and MMP-2 together dissect the Ln-5 γ2 chain into pro-migratory γ2 and γ2x fragments (103, 104). Therefore, in this situation, AQP3 appears to regulate the MMP enzymatic activity by modulating PI3K activity, which was first described in AQP5 (10). Similar positive correlations between the expression of AQP3 and MMP-2 or MMP-3 have been reported in lung cancer and prostate cancer cells (105, 106), and in this case, upregulation of MMP-3 seems to be related to ERK1/2 pathways, as has been reported for AQP5 (10).

AQP3 has been implicated in the EMT process. Induced expression of AQP3 in response to EGF in colorectal, gastric, and pancreatic cancer cell lines can enhance activities leading to the aggressiveness of growing cancer cells, including increased cell migration, invasion, and metastasis (107–109) with mesenchymal transformation (110). Expression of vimentin and fibronectin have also been found to be positively correlated with AQP3 expression, whereas E-cadherin expression has been shown to be significantly reduced among cancer cells with enhanced AQP3 expression.

AQP3, induced by EGF, can promote growth and cellular migration of various cancer cell lines through promoting H2O2 flux (109–112), which itself can trigger cell proliferation and migration (113, 114). Hydrogen peroxide produced by cell-surface NADPH Oxidase (Nox) enzymes is emerging as an important signaling molecule for growth, differentiation, and migration processes. However, how cells spatially regulate hydrogen peroxide to achieve physiological redox signaling over non-specific oxidative stress pathways is insufficiently understood. Principally, activation of various cell surface receptors activates Nox situated either in the plasma membrane or in the membrane of organelles such as endosomes to produce H2O2. To function as an intracellular signaling molecule, H2O2 must be imported into the cytosol. Cytosolic H2O2 enhances protein tyrosine phosphorylation by inactivating protein tyrosine phosphatases while activating protein tyrosine kinases. Transient protection of the H2O2signal from abundant cytosolic peroxiredoxin appears to result from the reversible inactivation of these enzymes through either hyperoxidation or phosphorylation. The suppression of AQP3 expression reduces EGF-induced H2O2 influx and attenuates EGF signaling cascades in various cancer cells (109, 112). Recently, Miller et al. (109) proposed that AQP3 promotes the delivery of H2O2 inside mammalian cells and promotes downstream intracellular signaling by H2O2. Novel imaging technology, a molecular imaging with Peroxy Yellow 1 Methyl-Ester (PY1-ME), a new chemoselective fluorescent indicator, has shown that enhanced expression of AQP3 and AQP8 can cause intracellular H2O2 accumulation. Importantly, AQP3 may be required for Nox-derived H2O2 signaling upon growth factor stimulation (115–117). Taken together, it seems that the downstream intracellular effects of H2O2 can be regulated across biological barriers at least partially by AQP3.

Of note, a report by Moon et al. (115) demonstrated the expression of AQP1, AQP3, and AQP5 in activated B and T lymphocytes, whereas none of the tested AQPs were expressed in inactivated B or T lymphocytes. Moreover, expression of AQP1, AQP3, and AQP5 was found among tumor-infiltrating lymphocytes surrounding bronchogenic carcinoma cells. It is important to note that H2O2 also influences chemokine-dependent migration of T-cells and that AQP3 might be involved in immune activation, at least partially, through H2O2 transport (116). Regulated T-cell migration and trafficking are of crucial importance for both steady-state T-cell homeostasis and active immune responses. Although naive T cells constitutively circulate between the blood and secondary lymphoid organs in a state of immune surveillance, antigen-encountered T cells selectively migrate to extralymphoid sites to exert their secondary response to antigens. The mechanistic basis of regulated T-cell trafficking involves the differential expression of adhesion molecules and chemokine receptors of naive and activated T cells. Chemokine-dependent trafficking is indispensable for the effector function of antigen-experienced T cells during immune responses. In this study, we report that AQP3 is expressed on T cells and regulates their trafficking in cutaneous immune reactions. T-cell migration toward chemokines is dependent on AQP3-mediated hydrogen peroxide uptake but not the canonical water/glycerol transport. AQP3-mediated hydrogen peroxide transport is essential for the activation of the Rho family GTPase Cdc42 and the subsequent actin dynamics. Coincidentally, AQP3-deficient mice are defective in the development of hapten-induced contact hypersensitivity, which is attributed to the impaired trafficking of antigen-primed T cells to the hapten-challenged skin. We therefore suggest that AQP3-mediated hydrogen peroxide uptake is required for chemokine-dependent T-cell migration in sufficient immune response.

In breast cancer (117), CXCL12/CXCR4-dependent cell migration plays an important role in progression, for which AQP3 is crucial through H2O2 transport *via* channel activity from AQP3. CXCL12/CXCR4-dependent cell migration is a critical process in breast cancer progression; however, its underlying mechanism remains to be elucidated. Here, in the report by et al., authors had demonstrated that CXCL12/CXCR4-dependent breast cancer cell migration that occurs through a mechanism involving extracellular hydrogen peroxide, produced by CXCL12-activated membrane Nox2, was transported into breast cancer cells *via* AQP3. Transient hydrogen peroxide accumulation was observed around the membrane during CXCL12-induced migration, which may be facilitated by the association of AQP3 with Nox2. Intracellular hydrogen peroxide then oxidized PTEN and protein tyrosine phosphatase 1B (PTP1B), resulting in the activation of the Akt pathway. This contributed to directional cell migration. Coincidentally, spontaneous metastasis of orthotopic xenografts to the lung was reduced upon AQP3 knockdown. These findings underscore the importance of AQP3-transported H2O2 in CXCL12/CXCR4-dependent signaling and migration in breast cancer cells and suggest that AQP3 has potential as a therapeutic target for breast cancer. Overall, several AQPs, including AQP1, AQP3, AQP4, AQP5, AQP8, and AQP9, have been suggested to transport H2O2, and their expression promotes cancer cell growth and migration (111, 118–127), However, solid data exist only for AQP3- mediated H2O2 transport as one of the key mechanisms for cancer cell migration.

The evidence presented from various report as discussed above indicates that some, but not all, AQPs can facilitate the uptake of H2O2 in mammalian systems and that the ability to mediate H2O2 transport may correspond to specific AQP classes (classical, aquaglyceroporin). Moreover, expression profiles of AQPs could dictate the susceptibility of a particular cell or tissue to external H2O2 signaling. Importantly, the notion that H2O2 uptake is controlled at biological barriers has broad implications for AQP channels as regulators of redox signaling, with potential connections in the areas of cell migration and wound healing. Moreover, AQP3 can be considered a target for T-cell–mediated diseases. For example, inflammatory skin diseases, such as atopic dermatitis and psoriasis, are characterized by the infiltration of T cells into both the dermis and the epidermis of affected skin, whereas different subsets of T cells are associated with each disease. Abundant T-cell infiltrates with AQP3 expression in the skin of the atopic dermatitis and psoriatic lesions, suggesting an important role of AQP3-mediated T-cell migration to the skin and novel therapeutic strategy for controlling unwanted immune reactions in the skin, including atopic dermatitis and psoriasis, and other autoimmune diseases can be expected in the future (116).

### Aquaporin 4

The role of AQP4 in human carcinogenesis has been largely focused on AQP4 as a water transporter in the brain as it was initially discovered as brain-specific water channel. However, it has recently been suggested that AQP4 also plays an important role in brain tumor development and progression, although these studies are still in the early stage. AQP4 is predominantly expressed in the brain astrocytes, foot processes surrounding blood vessels. From the study of AQP4 KO mice, two common brain pathologies, pseudotumor cerebri and cerebral edema (126–128), were hypothesized to involve AQP4 in their pathophysiology. AQP4-null mice were shown to be resistant to water intoxication, meningitis, and brain ischemia (129, 130) and carry a significantly augmented brain water content with increased intracranial pressure compared with wild-type mice (130, 131). AQP4 can control bidirectional water flux and may therefore be responsible for brain edema, while expression of AQP4 counteracts vasogenic edema (128). In the last decade, the reclassification of edema as cytotoxic, ionic, or vasogenic based on the observed changes in the brain has been widely adopted (132). In this sense, although AQP4 can play a protective role in brain swelling by increasing brain water clearance, in cytotoxic edema, AQP4 is the main contributor to astrocytic cell swelling (129–133). Indeed, water-intoxicated AQP4-null mice showed reduced astrocytic foot process swelling and decreased brain water content (134). This dual function of AQP4 may play an important role in any processes involving brain swelling, including brain tumors, which may be extended to metastatic tumors to the brain. For example, melanoma has a high propensity to invade the brain and often causes significant peritumoral edema. Indeed, in mice models, melanoma cells implanted into the striatum of wild-type and AQP4-null mice produced edema surrounding the tumor mass and comparable sized-tumors in both groups after a week. Surprisingly, the AQP4-null mice had a higher intracerebral pressure and water content (129, 130), which are often observed in terminal cancer patients with brain metastasis and patients who undergo radiation therapy (129–131). These findings may help to design an AQP4 modifier for the control of brain edema for terminal patients with brain metastasis who often suffer serious clinical symptoms associated with brain edema.

The expression of AQP4 has been shown to be upregulated in certain forms of astrocytoma, meningioma, and metastatic tumors (131–136). For example, AQP4 expression was shown to be increased in glioblastoma multiforme (GBM), which is often accompanied by AQP4 redistribution in glioma cells (131). In the initial report by Warth et al., based on 189 WHO grade I–IV gliomas, significant expression of AQP4 was demonstrated with prognostic significance (133). In gliomas, a redistributed expression of AQP4 was observed compared with normal central nervous system tissue. Indeed, the highest membranous staining levels were seen in pilocytic astrocytoma, WHO grade I, which carries a better prognosis than grade IV glioblastomas, which carries the worst prognosis.

AQP4 expression and VEGF-VEGFR-2 expression have been shown to be closely correlated, as were seen for AQP1 (40, 131–134). Moreover, it has been demonstrated that reduced expression of AQP4 parallels with VEGF-VEGFR-2 expression. Of note, in the peripheral areas of relapsed tumors, AQP4 expression assumed normal findings of a perivascular expression pattern. Although the interpretation of this observation needs to be examined in larger samples, in GBM, chemotherapy and radiotherapy downregulate AQP4 expression and can restore its perivascular rearrangement (131). Moreover, it has been suggested that a polarized rearrangement of AQP4 expression in peri-tumoral area specimens after combined chemotherapy and radiotherapy can lead into the normalization of tumor blood vessels. Still, the clinical meaning of this observation remains unclear. On the basis of expression profiles of brain tumor and its normal expression in astrocytes, in addition to the distinctive patterns of chemo and radiation of therapy, the strategy of designing AQP4-targeted therapy can be challenging. Although we hope to improve tumor-associated edema and secondary edema associated with chemoradiation therapy, which is usually treated with high dose of steroids, we expect that antagonizing AQP4 in GMB will result in secondary brain edema and that any approach targeting the activity of AQP4 in GBM may require continuous steroid treatment.

Tumor implantation experiments in AQP4-null mice have further solidified the role of AQP4 in the development of glioma. McCoy et al. (134) recently provided an initial mechanistic insight and merits further discussions. In this report, using glioma cells stably transfected with either AQP1 or AQP4, it was clearly demonstrated that PKC activity regulates water permeability and that this phenomenon can be modulated by phosphorylation of AQP4. It is important to note that glial-derived tumors, gliomas, are highly invasive cancers that invade normal brain through the extracellular space. Two members of this family, AQP1 and AQP4, a have a consensus phosphorylation site for PKC, which is a known regulator of glioma cell invasion. AQP4 colocalizes with PKC to the leading edge of invading processes and clustered with chloride channel and K(+)-Cl(−) cotransporter 1 (KCC1), inferred to provide the pathways for Cl(−) and K(+) secretion to accomplish volume changes. Using D54MG glioma cells stably transfected with either AQP1 or AQP4 expression construct, it was further demonstrated that PKC activity regulates water permeability through phosphorylation of AQP4. Activation of PKC with either phorbol 12-myristate 13-acetate or thrombin enhanced AQP4 phosphorylation, reduced water permeability, and significantly decreased cell invasion. Conversely, inhibition of PKC activity with chelerythrine reduced AQP4 phosphorylation, enhanced water permeability, and significantly enhanced tumor invasion. PKC regulation of AQP4 was lost after mutational inactivation of the consensus PKC phosphorylation site S180A. Interestingly, AQP1 expressing glioma cells, by contrast, were completely unaffected by changes in PKC activity. To demonstrate a role for AQPs in glioma invasion *in vivo*, cells selectively expressing AQP1, AQP4, or the mutated S180A-AQP4 were implanted intracranially into SCID mice. AQP4 expressing glioma cells showed significantly reduced invasion compared with AQP1 and S180 expressing tumors as determined by quantitative stereology, consistent with a differential role for AQP1 and AQP4 in this process.

A role for AQP4 in glioma cell migration was initially proposed to occur through the regulation of cell volume and cytoskeletal interactions (137). As discussed above, PKC-mediated phosphorylation of AQP4 at serine 180 correlated with a decreased glioma cell invasion (134, 138). The activation of PKC by two known PKC activators enhanced AQP4 phosphorylation, resulting in the reduced water permeability with significantly decreased tumor cell invasion. Likewise, the inhibition of PKC activity reduced AQP4 phosphorylation with enhanced water permeability and tumor cell invasion. However, these data seem to be contradictory to the observation made by Verkman et al., who reported that improved tumorigenicity by PKC activation depends on the use of a skin carcinogenesis model (91). We surmise that as the origin of skin epithelial cells and astrocytes are different, the PKC dependency of AQP4 may be dependent on voltage-dependent channels expressed in astrocytoma and GBM. Recent *in vitro* and *in vivo* experiments have demonstrated that AQP4 expression can control the potential for migration and invasiveness of glioma cell lines through a mechanism involving MMP2 (139). AQP4 increases the expression and localization of (ClC2) and the potassium-chloride cotransporter 1 in invadopodia, resulting in water efflux and secondary cell shrinkage; this series of events may be vital for the movement of tumor cells through the ECM (140).

AQP4 interacts with a-syntrophin through the C-terminal domain at a PDZ-binding site at the end foot of astrocytes. AQP4 is connected to the PSD95-PDZ domain of syntrophin, a component of the dystrophin protein complex (137), which promotes polarized subcellular localization of AQP4, and this study merits some detailed discussion. According to report by Neely et al., the authors have postulated that AQP4 is tethered at this site by binding of the AQP4 C terminus to the PSD95-Discs large-ZO1 (PDZ) domain of syntrophin, a component of the dystrophin protein complex. Chemical cross-linking and coimmunoprecipitations from brain demonstrated AQP4 in association with the complex, including dystrophin, beta-dystroglycan, and syntrophin. AQP4 expression was studied in brain and skeletal muscle of mice lacking alpha-syntrophin [alpha-Syn(−/−)]. The total level of AQP4 expression appears normal in brains of alpha-Syn(−/−) mice, but the polarized subcellular localization is reversed. High-resolution immunogold analyses revealed that AQP4 expression is markedly reduced in astrocyte end feet membranes adjacent to blood vessels in cerebellum and cerebral cortex of alpha-Syn(−/−) mice but is present at higher than normal levels in membranes facing neuropil. In contrast, AQP4 is virtually absent from skeletal muscle in alpha-Syn(−/−) mice. Importantly, the deletion of the PDZ-binding consensus (Ser-Ser-Val) at the AQP4 C terminus similarly reduced expression in transfected cell lines, and pulse-chase labeling demonstrated an increased degradation rate. These results demonstrate that perivascular localization of AQP4 in brain requires alpha-Syn, and the stability of AQP4 in the membrane is increased by the C-terminal PDZ-binding motif.

In experiments using glioma cell lines and a primary astrocyte model, reduced expression of AQP4 was shown to result in reduced cell migration and compromised F-actin formation (139, 140). Of note, the suppression of AQP4 in a mouse astrocyte culture model results in downregulation of connexin43, with a concomitant reduction in cell-to-cell electrical coupling (140, 141), indicating a potential functional relationship between AQP4 and gap junctions in brain astrocytes. Importantly, Binder et al. have reported the role of AQP4 in the maintenance of neuronal excitability. Although glial water channel AQP4 has been hypothesized to modulate water and potassium fluxes associated with neuronal activity, a dependable *in vivo* animal model was lacking till this report is published, which examined the seizure phenotype of AQP4 −/− mice using *in vivo* electrical stimulation and electroencephalographic (EEG) recording. AQP4 −/− mice were found to have dramatically prolonged stimulation-evoked seizures after hippocampal stimulation compared with wild-type controls. In addition, AQP4 −/− mice were found to have a higher seizure threshold. To assess a potential effect of AQP4 on potassium kinetics, reading from potassium-sensitive microelectrodes after direct cortical stimulation. Although there was no significant difference in baseline or peak [K(+)](o), the rise time to peak [K(+)](o) and the recovery to baseline [K(+)](o) were slowed in AQP4 −/− mice compared with WT mice. These results implicate AQP4 in the expression and termination of seizure activity and support the hypothesis that AQP4 is coupled to potassium homeostasis *in vivo*.

The increase in AQP4 expression and redistribution/surface localization are two concepts. Previous studies have shown an increase in AQP4 membrane localization in primary human astrocytes, which was not accompanied by a change in AQP4 protein expression levels. Although the mislocalization of AQP4 was proposed to be a mechanism of alleviating benign disease, such as autoimmune mediated CNS pathophysiology, it may also be useful for designing anticancer therapeutic targets (141–143). In this sense, the report by et al. merits detailed discussion (142). In this report, using a combination of single-molecule and calcium imaging approaches, in the hippocampal astrocytes, the dynamic distribution of the AQP4 isoforms M1 and M23 was investigated. AQP4 exists in two main isoforms, M1 and M23, which exhibit similar water transport capacities but different aggregation properties and cellular distribution onto astrocytes. At the structural level, the two isoforms have similar extracellular domains but different intracellular ones. In addition, these two isoforms have different surface- trafficking characteristics in pure cultured astrocytes. Thus, the possibility that AQP4 isoforms differentially control astrocytic process motility, through distinct membrane organization, has emerged and gained support. Surface AQP4-M1 formed small aggregates that contrast with the large AQP4-M23 clusters that are enriched near glutamatergic synapses. Strikingly, stabilizing surface AQP4-M23 tuned the motility of astrocyte processes and favors glutamate synapse activity. Furthermore, human autoantibodies directed against AQP4 from patients with neuromyelitis optica (NMO) impaired AQP4-M23 dynamic distribution and, consequently, astrocyte process and synaptic activity. Collectively, it emerges that the membrane dynamics of AQP4 isoform regulate brain cell assemblies in health and autoimmune brain disease targeting AQP4.

Importantly, the two most recent studies have demonstrated the important role of AQP4 in generating CNS edema following injury-induced hypoxia (144, 145). The study by Kitchen et al. demonstrated that ischemia and CNS edema are associated with increases in both total AQP4 expression and AQP4 subcellular translocation to the blood–spinal cord-barrier (BSCB) (144). Importantly, pharmacological inhibition of AQP-4 translocation to the BSCB helps to treat ischemia- induced CNS edema and promotes functional recovery in injured rats (144). This role has been confirmed by the work of Sylvain et al., which has demonstrated that targeting astrocytes effectively reduces cerebral edema during the early acute phase of stroke using a photothrombotic stroke model (145). A link to brain energy metabolism has also been demonstrated, as indicated by the increase in glycogen levels. Both of these studies provide a biological basis for designing AQP4 modulating agents for treating ischemia-induced CNS edema, which may also alleviate various neurological manifestations among patients with brain tumors.

Various AQPs have been linked to EMT in different types of cancer cells. In lung adenocarcinoma cells, AQP1 overexpression correlated with the downregulation of E-cadherin and the upregulation of vimentin (82). The suppression of AQP4 in a human breast cancer cell line model upregulated E- cadherin, whereas in glioma cells, it resulted in enhanced β-catenin and connexin 43 expression (139–141, 146). Moreover, in primary human astrocytes, reduced expression of AQP4 resulted in downregulation of connexin-43 (140).

### Aquaporin 5

**Figure 3:**
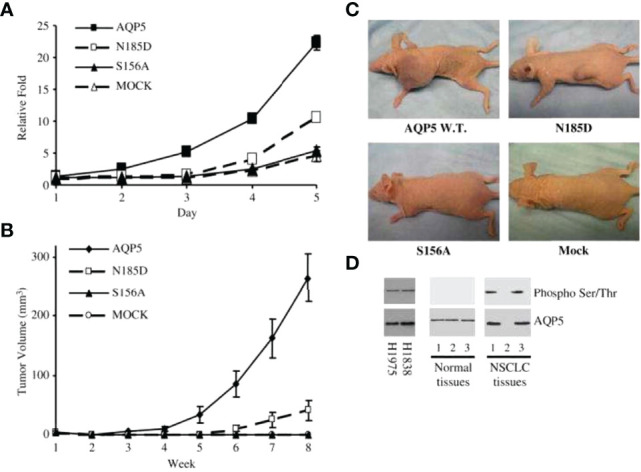
AQP5 increases cell proliferation, which depends on Ser156 phosphorylation dependent. **(A)** Cell proliferation is significantly increased in stable cells with wild-type AQP5 or the N185D mutant as compared with cells with the S156A mutant or the control vector. **(B, C)** Tumor growth in athymic mice. Significant tumor growth was detected within 8 weeks after subcutaneously injected to athymic mice model with stable cells with wild-type AQP5 or the N185D mutant. No tumor growth is detected in mice injected with stable cells with the S156A cells or MOCK **(C)**. **(D)** Phosphorylation of AQP5 in cancer cells and resected lung cancer cells. AQP5 is phosphorylated in the two lung cancer cell lines (H1975 and H1838), and in two of three primary non–small cell lung cancer tissue samples. Such phosphorylation is not detected in three primary normal tissues. Adapted from (148).

Increased expression of AQP5 was initially reported in colon and pancreatic cancers (26, 49, 147). During colorectal carcinogenesis, the expression of AQP1 and AQP5 was induced in early-stage disease (early dysplasia) and maintained through the late stages of colon cancer development (**Figure 1**) (26). These observations led us to study the molecular mechanisms behind AQP5-induced oncogenesis. In a report by Woo et al., as described in **Figure 3**, the overexpression of AQP5 in NIH3T3 cells demonstrated a significant activation of the Ras pathway, and activation of Ras was shown to be mediated by the phosphorylation of the PKA consensus site of AQP5 (10, 124, 148, 149). This is the first evidence demonstrating an association between AQP and Ras signal transduction pathway and serves as a basis for the oncogenic properties of AQP. Another study by Woo et al. demonstrated that human AQP5 (hAQP5) in BEAS-2 cells induces many phenotypic changes (**Figure 3**) and that this effect of AQP5 depended upon the phosphorylation of a cAMP–protein kinase (PKA) consensus site located in a cytoplasmic loop of AQP5 (10, 124, 148, 149). In addition, PKA consensus site phosphorylation was found preferentially in resected tumor samples compared with normal counterparts. Likewise, Kang et al. (149) conducted a more focused study in colorectal carcinogenesis. As seen in NIH3T3 and BEAS-2 cells, overexpression of wild-type hAQP5 increased the proliferation and phosphorylation of extracellular signal-regulated kinase-1/2 in HCT116 colon cancer cells. Colon cancer cells with hAQP5-overexpressing constructs showed an increase in retinoblastoma protein phosphorylation through the formation of a nuclear complex with cyclin D1 and CDK4. These data provided a unique molecular mechanism for colon cancer development through the interaction of hAQP5 with the Ras/extracellular signal-regulated kinase/retinoblastoma protein signaling pathway, extending the role of AQP5 expression in cell cycle regulation.

In the case of lung cancer, Chae et al. (124) reported that, among more than 400 resected non–small cell lung cancer samples, various degrees of AQP5 expression have been observed with significant prognostic implications. BEAS-2B and NIH3T3 cells stably transfected with constructs wild-type AQP5 (AQP5) and two mutants, N185D, which prevents membrane trafficking, and S156A, which prevents phosphorylation on Ser156, showed that only wild-type AQP5 caused cell invasion in an *in vitro* cell migration assay. Moreover, only the expression of wild-type AQP5 caused full morphological changes from EMT, which included transformation into spindle-like cellular shapes and fibroblastic morphologic changes, with reduced cell–cell contacts and cell polarity. Only BEAS-2B cells that express wild-type AQP5 exhibited a loss of epithelial cell markers, such as E-cadherin, α catenin, and γ catenin, and a gain of mesenchymal cell markers, including fibronectin and vimentin (23, 146, 150). AQP5 demonstrated robust binding activity to SH3-domains of the c-Src, Lyn, and Grap2 C-terminal. Importantly, these observations suggest a potentially crucial role of AQP5 as a tyrosine kinase activator, which may induce the initial stage of EMT. In addition, this report by Chae et al. and other follow-up studies discussed below suggest that the mechanism underlying AQP5-facilitated cancer cell invasion and metastasis is due to its direct or indirect interaction with EGFR/ERK1/2.

A later follow-up study reported that AQP1, AQP3, and AQP5 were detected in 71%, 40%, and 56% of resected lung cancers, respectively. Similar to the report by Chae et al., the follow-up found that the expression of AQPs was more frequently seen among adenocarcinomas, and the expression of AQP1 and AQP5 was minimal in squamous cell carcinomas (151–154). Expression of AQP1, AQP3, and AQP5 was negatively associated with the degree of cellular differentiation, and higher expressions of AQP1 and AQP5 were observed among invading lung cancer cells with metastasis.

Similarly, from several reports using resected pancreatic ductal adenocarcinoma and cell lines, AQP3 and AQP5 overexpression was found to be associated with reduced expression of E-cadherin and increased levels of vimentin (124, 146, 150–154). The potential for the invasion-promoting properties of AQP5 expression was at least partially dependent on the c-Src signaling pathway and knowing the role of c-Src as a mediator of EMT (124, 146, 148–150). Other studies using different cancer cell lines and resected cancer tissue samples demonstrated that AQP5 expression was correlated with lymph node metastasis in patients with colorectal cancer (152), the proliferation and migration of human breast cancer cells (153), and the metastatic potential of lung cancer cells (154).

High AQP5 expression can enhance the level of phosphorylated SMAD2, promoting EMT in a colon cancer cell model, whereas suppressed expression of AQP5 can downregulate phosphorylated SMAD2, resulting in downregulated EMT response (155). The activity of c-Myc, which plays a key role during the cell cycle as a potent oncogene and a major cell cycle modifier, can be modified by AQP5 (155–157). Moreover, AQP5 enhanced the activation of Src-kinase (146), a key modifier of Smad2 phosphorylation, during TGF-beta-induced EMT, which warrants further study.

LGR5 marks resident adult epithelial stem cells at the gland base in the mouse pyloric stomach (157 144), and LGR5+ intestinal stem cells are major sources of cancer cell generation following hyperactivation of the WNT pathway (156, 157), a key regulator of stem cell behavior (157–160). Axn2, a WNT-regulated protein, is located in the base of gastric glands, and its expression is mainly limited among Lgr5+ cells and actively dividing Lgr5− cells. The pathways from LGR5+ stem cells to the fully developed intestinal epithelia or cancerous tissues are not clearly understood (156, 157).

In a report by Tian et al., a Lgr5 knock-in mouse model and a Lgr5-eGFP-IRES-creERT2 mouse model were used to profile the transcriptional landscape of LGR5+ stem cells (160) by microarray, and six candidate genes including AQP5 were identified as markers for stem cell activity. Additional experiments have demonstrated that, compared with AQP5− cells, AQP5+ cells could generate long- term passage organoids. Importantly, AQP5 expression was accompanied by MUC6, A4GNT, and SLC9A3 expression, all of which coincided with LGR5 expression. Further experiments using human AQP5+ cells confirmed that AQP5 is key protein enabling organoid generation that can be passaged over 3 months. A second *in vivo* mouse model (Aqp5-IRES-creERT2 APK) study for comparative profiling of LGR5+ stem cell populations along the gastrointestinal tract confirmed the stem potential of tumor-initiating cells within the AQP5+ compartment. Finally, stem cells within the AQP5+ compartment may represent an initiating point of WNT-driven, invasive gastric cancer (158).

The report by Tian et al., for the first time, provided convincing evidence for the role of AQP5+ cells in mouse models of gastric carcinogenesis. Of note, the expression of MUC6, which is enriched among AQP5+ cells, was unexpected given that, in an undifferentiated stem cell model, MUC6 expression should not be located among stem cell populations. Some recent data suggest that even more defined cells can provide as a key stem cell function (161–170). Overall, the recent report by Tian et al. will be an invaluable resource for studying the molecular pathways, leading to the development of gastric cancer and possibly other solid tumors

Of note, our group has initially described novel roles of AQP5 in cancers as a driver of proliferation and invasiveness *in vitro* and its expression in human resected cancer samples (10, 124, 146, 148, 149, 171). Since these reports, a series of similar reports analyzing resected tumor samples from gastric, breast, soft tissue sarcoma, lung, esophageal, and colorectal cancers have demonstrated various degrees of AQP expression. Moreover, the proliferation and invasive potential of AQPs have been demonstrated in multiple cancer cell lines (172–176). Whereas prior biochemical pathways have suggested a potential role of AQP5 as a key marker for the early stage of human carcinogenesis, a series of observations by Tian et al. provided confirmatory evidence for the role of AQP5 in the initiation of gastric carcinogenesis and as a crucial player of cancer stem cell activity.

### Aquaporins 9, 8, and 2

Robust expression of AQP9 in the cellular membrane has been observed in various brain tumors, and it was further proposed that the clearance of glycerol and lactate by AQP9 can alleviate lactic acidosis from high lactate-producing cancer cells (177, 178). In prostate cancer cells, AQP9 could enhance ERK1/2 and MMP9 signaling (178). As discussed above, an initial study demonstrated that AQP3 is required for (NOX)-derived H2O2 signaling, while AQP8 can transport NOX-generated H2O2 (177, 179–186) and AQP8 expression induced by EGF can promote cellular intake of H2O2, resulting in a series of pathways leading into tyrosine phosphorylation of multiple signaling proteins (187).

H_2_O_2_ must cross a membrane to reach its cytosolic targets. H_2_O_2_ has long been thought to cross lipid bilayers freely. However, the slightly larger dipole moment of H_2_O_2_ makes its simple diffusion through membranes less likely than for water. Its size and electrochemical properties qualify H_2_O_2_ as a possible substrate of aquaporins (AQP). In this sense, report by Bertolotti et al. merits further discussion. In this report, silencing aquaporin-8 (*AQP8*), but not *AQP3* or *AQP4*, inhibited H_2_O_2_ entry into HeLa cells. Re-expression of AQP8 with silencing-resistant vectors rescued H_2_O_2_ transport, whereas a C173A-AQP8 mutant failed to do so. Lowering AQP8 levels affected H_2_O_2_ entry into the endoplasmic reticulum, but not into mitochondria. *AQP8* silencing also inhibited the H_2_O_2_ spikes and phosphorylation of downstream proteins induced by epidermal growth factor. These observations lead to the hypothesis that H_2_O_2_ does not freely diffuse across the plasma membrane and AQP8 and other H_2_O_2_ transporters are potential targets for manipulating key signaling pathways in cancer and degenerative diseases.

AQP8- mediated H2O2 transport has been suggested as a vital component of B lymphocyte activation (179) and leukemia cell proliferation (180, 181). Importantly, role of hydrogen peroxide as a second messenger and one of key signaling circuit controller, modulating tyrosine phosphatases and kinase activity, during B cell activation and differentiation have been described by et al. In this report, NADPH-oxidase 2 was identified as the main source of H2O2 and aquaporin 8 as a transport facilitator across the plasma membrane. Upon aquaporin 8 silencing, inducible B lymphoma cells responded poorly to TLR and BCR stimulation. Their differentiation was severely impaired, as demonstrated by retarded onset of IgM polymerization, low amounts of IgM secretion, and prolonged BCR expression on the cell surface. A silencing-resistant aquaporin 8 rescued responsiveness, confirming that the import of H2O2 across the membrane is essential for B cell activation. The addition of exogenous catalase to primary B splenocytes severely impaired the tyrosine phosphorylation induced by BCR cross-linking, as did the absence of NOX2 in a murine model of chronic granulomatous disease. Importantly, re-expression of gp91phox through gene therapy restored the specific B cell signaling deficiency in NOX2-/- cells. Thus, efficient induction of B cell activation and differentiation requires intact H2O2 fluxes across the plasma membrane for signal amplification.

AQP8 is exclusively expressed on the bile canaliculi of the liver (182, 183) and it was further suggested that AQP8 plays an important role in the homeostasis of H2O2 produced by the sinusoidal cells from NADPH oxidase complex. AQP8 is also expressed in the inner mitochondrial membranes of hepatocytes (182–184). In the human hepatocyte carcinoma cell line HepG2, mitochondrial AQP8 (mtAQP8) seems to play a key role during the efflux of H2O2 generated from various metabolic processes (183). In mitochondrial AQP8b, the marine testis homolog of human AQP8 (185, 186), involvement of mtAQP8-mediated H2O2 transport seems to be vital for proper function of spermatozoa, while a similar observation has been made during human spermatogenesis (187). Suppression of HepG2 mtAQP8 expression dramatically reduces the release of mitochondrial H2O2 with the accumulation of mitochondrial ROS, which can result in compromised production of ATP (185). ROS has been shown to compromise mitochondrial activity, resulting from the suppression of AQP8, which can cause apoptotic and necrotic death of various cells, including HepG2 cells (185, 188, 189). Of note, aquaporin-8 (AQP8) allows the bidirectional transport of water and hydrogen peroxide across biological membranes and H_2_O_2_ exerts opposite roles, amplifying growth factor signaling in physiological conditions, but causing severe cell damage when in excess and this process is based on tight control of H_2_O_2_ permeability is likely to be rigrously controlled in living cells. Recently, report by Fernandez et al. has investigated whether and how the transport of H_2_O_2_ through plasma membrane AQP8 is regulated, particularly during cell stress (189). This study shows that diverse cellular stress conditions, including heat, hypoxia, and ER stress, reversibly inhibit the permeability of AQP8 to H_2_O_2_ and water. Preventing the accumulation of intracellular reactive oxygen species (ROS) during stress counteracts AQP8 blockade. Once inhibition is established, AQP8-dependent transport can be rescued by reducing agents. Neither H_2_O_2_ nor water transport is impaired in stressed cells expressing a mutant AQP8, in which cysteine 53 had been replaced by serine. Cells expressing this mutant are more resistant to stress-, drug-, and radiation-induced growth arrest and death. Severe stress is followed by growth arrest and cell death, which limits proliferation of damaged or transformed cells. Stresses most often induce intracellular production of ROS. In this scenario, blocking AQP8-dependent transport can provide a dual defense against oxidative damage: if the stress is mild, cells resume growth once the problem is solved; if it is harsh, cells undergo apoptosis, limiting the deleterious effects of ROS at the organism level. Cells expressing the noninhibitable AQP8 C53S variant are more resistant to stress-induced growth arrest and ROS-triggered apoptosis, thus increasing survival. Stress-induced inhibition of water transport may also contribute to the cell responses to stresses. This study, from various different conditions causing cellular stress and degree of stress, conclude that the control of AQP8-mediated H2O2 transport can provide a novel mechanism to regulate cell signaling and survival during stress.

The RGD motif of AQP2 promotes cell migration by connecting integrin β1 activity to focal adhesion sites (190, 191). When AQP2 is present, integrin β1 can be recycled out of the focal adhesion complex, AQP2 can promote recycling of focal adhesions, and this activity of AQP2 can facilitate cell migration. Similar mechanisms were used by AQP2 in estrogen-activated endometrial carcinoma cells by binding to annexin-2 and integrin β1, which can enhance proper time- and location-dependent F-actin reorganization (192). Report by Zou et al. has demonstrated that AQP2 was expressed in endometrial tissues from patients with endometrial cancer and endometriosis, both of which are estrogen-dependent diseases. In IshikawaIshikawa (IK) cells, E(2) dose-dependently increased AQP2 expression, which was blocked by the estrogen receptor inhibitor ICI182780. An estrogen-response element was identified in the AQP2 promoter. E(2) significantly increased the migration, invasion, adhesion, and proliferation of IK cells. AQP2 knockdown reduced not only the E(2)-enhanced expression of F-actin and annexin-2 but also the E(2)-induced alteration of cell morphology. Moreover, higher expression levels of F-actin and annexin-2 were detected in the endometrial tissues from patients with EC. However, AQP2 can be imported to the nuclei when glioma cells are treated with estrogen. Therefore, it can then be complexed with estrogen nuclear receptors (ERα and ERβ) and these binding activity of AQP2 can suppress cell invasion (193).

Of note, several studies have shown that single amino acid substitutions in the selectivity filters of AQP1, AQP4, and AQP3 differentially affect glycerol and urea permeability in an AQP-specific manner (194, 195). Comparison between silico-calculated channel cross-sectional areas and *in vitro* permeability measurements suggests that the selectivity filter cross-sectional area predicts urea but not glycerol permeability (194). Recent data show that substrate discrimination in water channels depends on a complex interplay between the solute, pore size, and polarity, and that using single water channel proteins as representative models has led to an underestimation of this complexity (194, 195). Therefore, single amino acid substitution experiments for various AQPs are likely to lead to better understanding of the diverse function of AQPs during mammalian carcinogenesis.

### Aquaporins 11, 7, 6 and 0

AQP11 is highly expressed in testis and, to a lesser degree, in kidney, liver, brain, and adipose tissue (196). AQP11 functions as an ER-resident peroxiporin, and its downregulation perturbs the flux of H2O2 through the ER while it does not affect flux of H2O2 through the mitochondrial or plasma membranes (17). AQP11 mRNA was upregulated during adipocyte differentiation, and proinflammatory factors TNF-α, and particularly TGF-β1, were downregulated AQP11 mRNA (197).

An initial report by Evans et al. has suggested that high AQP11 expression can be associated with reduced overall survival among patients with lung adenocarcinoma (198). Cell line experiment suggested that, among eleven cancer cell lines with AQP11 expression, there was a significant association of AQP11 expression with cisplatin resistance. Furthermore, high AQP11 level seems to play a role as one of the pro-survival factors, protecting tumor cells from cisplatin-induced stress. The Cancer Genome Atlas (TCGA) database analysis of previously untreated lung adenocarcinoma detected 13% tumors with elevated AQP11 mRNA expression. Increased AQP11 expression was significantly associated with decreased overall survival. These patients showed lower median survival rate of 34.47 versus 52.5 months in patients with low AQP11 expression. A follow-on study using matched mRNA expression, survival, and drug response data from TCGA lung adenocarcinoma dataset (N = 369), In this report, Sharpnack et al. have demonstrated that high AQP11 mRNA expression is negatively prognostic in patients with adenocarcinoma of lung (LUAD) (199). Furthermore, patients with high tumor AQP11 mRNA expression (10.3%) had significantly worse overall survival compared with patients with low AQP11 expression. An analysis of patients treated with platinum-based chemotherapy (N = 74) showed that patients with high AQP11 mRNA expression (7%) had significantly higher overall survival then patients with LUAD expressing low levels of AQP11 Both reports suggested that, with further validation, AQP11 level might be a predictor of cisplatin resistance and overall survival in lung cancer.

AQP7 (human AQP7/mouse Aqp7) is a transmembrane aquaglyceroporin and member of the aquaporin family (200). AQP7 also transports hydrogen peroxide, ammonia, urea, and arsenite (200–205). Recently, a study for the integration of metabolomics and gene expression data from breast cancer mouse models was reported and this study identified 35 metabolite and 34 gene hubs with the most network correlations, which carry the prognostic value and are integral to tumor metabolism and breast cancer (206). Unexpectedly, the gene hub Aquaporin-7 (Aqp7) was identified as a novel regulator of breast cancer and that AQP7 was prognostic of overall survival in patients with breast cancer. In mouse breast cancer models, reduced expression of Aqp7 caused reduced primary tumor burden and lung metastasis (206). Metabolomics and complex lipid profiling of cells and tumors with reduced Aqp7 revealed significantly altered lipid metabolism, glutathione metabolism, and urea/arginine metabolism compared with controls. These data identify AQP7 as a critical regulator of metabolic and signaling responses to environmental cellular stresses in breast cancer, highlighting AQP7 as a potential cancer-specific therapeutic vulnerability.

Studies for the role of AQP6 in mammalian carcinogenesis have been largely lacking other than one report regarding AQP6 expression in ovarian tumor (207). In this report by Ma et al., the expression of AQP6 and AQP8 from 47 cases of epithelial ovarian tumors was examined by immunochemical technique and Western blotting. AQP6 was strongly expressed in benign ovarian tumors, but weak signal was shown in malignant tumors (207). The difference was not statistically significant.

Compared with serous adenoma and normal tissues, AQP6 expression in serous carcinoma was obviously decreased.

AQP0 mRNA expression has been identified in retina, liver, and Sertoli cells of testis and abundantly expressed in the lens fiber cells (208). Moreover, Shen et al. (209) found low AQP0 mRNA expression in human gastric carcinoma and corresponding normal tissue through RT-PCR method. Studies about the correlation of AQP0 mRNA with gastric cancer, based on the database study, that AQP0 mRNA was associated with poor survival rate in all cancer patients, especially with in testinal type both in male and female, and as well as in clinical stages I and III (210).

### Summary of Overall Development of AQP Targeting Therapeutics

Although it was initially proposed as an attractive anticancer therapeutic, designing clinically relevant AQP-directed anticancer therapeutics is an early stage and still lacks clinical trial with many difficulties in the way. So far, AQP-target inhibitors, including cysteine-reactive heavy metal–based inhibitors, small-molecule inhibitors that inhibit AQPs expression or AQP-induced water permeation, and monoclonal AQP-specific antibody, have been developed and verified. Of note, AQP gene transfer has been developed and applied to clinical therapy that eleven participants with previously irradiated parotid glands after radiation therapy for head or neck cancer received AQP1-cDNA transfer therapy in the phase I clinical trial (211), whereas the efficacy and safety of AQP gene transfer warrants future studies. Initial observation that mercury can block the water-transport function in AQPs with Cys 189, such as AQP1 resulted in the development of heavy metal–based AQPs inhibitors such as silver-, gold-, or ruthenium-containing compounds as potential anticancer drugs (212, 213). In fact, the common chemotherapy drug cisplatin was shown to inhibit the expression of AQP5 which is upregulated in ovarian tumor and associated with lymph node metastasis and ascites (214). Several small-molecule compounds without heavy mental were shown to suppress tumorigenic effects of AQPs. Acetazolamide, carbonic anhydrase inhibitor, was found to inhibit the expression of AQP1, which protected tumor from cytotoxic edema by maintenance of extracellular acidification and promoted tumor metastasis in glioma (215). Moreover, several AQP1- target inhibitors [including cyclophosphamide, topiramate, and anesthetic drugs (216)], AQP4-target inhibitors including anti-epileptic drugs, bumetanide, sumatriptan and thiadiazole (217). and AQPs-target inhibitors [including TEA+ (77)] were tested in several cancer cell line models with various degree of cytotoxicity.

The AQP4-IgG detected in patients with NMO is specially bound to AQP4 and inhibits AQP4 water permeability, thereby leading to complement-dependent cytotoxicity in astrocytes (218). Now, the monoclonal AQP4-specific antibody has been developed (219). Thus, the speculation that AQP4- specific antibody linked with toxin can be used to selectively damage AQP4-expressing glioblastoma cells has been proposed (217).

In summary, although detailed discussion for each of AQP targeting anticancer therapeutics is beyond the scope of this review. The process in AQP-target inhibitors development has also been slow and so far, none of lab-developed AQP targeting drugs has been applied for clinical anticancer therapy by far, not to mention reaching to the stage of clinical trial.

### Aquaporin Genomic Instability and Binding Partners

Hanahan and Weinberg reported six functional capabilities of cancers, described as hallmarks of cancer (12, 23). These six hallmarks include self-sufficiency in growth signals, insensitivity to anti- growth signals, evasion of apoptosis, sustained angiogenesis, tissue invasion and metastasis, and unlimited replicative potential. Since the initial discerption, an additional concept has been introduced, which describes how mutations lead to these hallmarks. As discussed below, genomic instability was considered a separate but crucial step as it enables the acquisition of hallmarks. In other words, genomic instability is a property driving tumorigenesis.

In the case of AQPs, numerous published datasets, extensively discussed above, suggest that AQPs play an important role in certain aspects of the six hallmarks, converting normal cells to tumor cells and changing the behavior of tumor cells. In this regard, we propose that AQP expression can change the behavior of normal cells, potentially by enhancing genetic instability, which might result in unrestricted cellular growth. Further mechanistic understanding of the role of AQPs during the development of these six hallmarks and the establishment of genetic instability in cancer cells will be crucial for better management of patients with various types of cancer. Moreover, we expect to see the “clinical validation study” for the role of novel AQP antagonists in the management of various human cancers (64, 66, 74).

**Figure 4:**
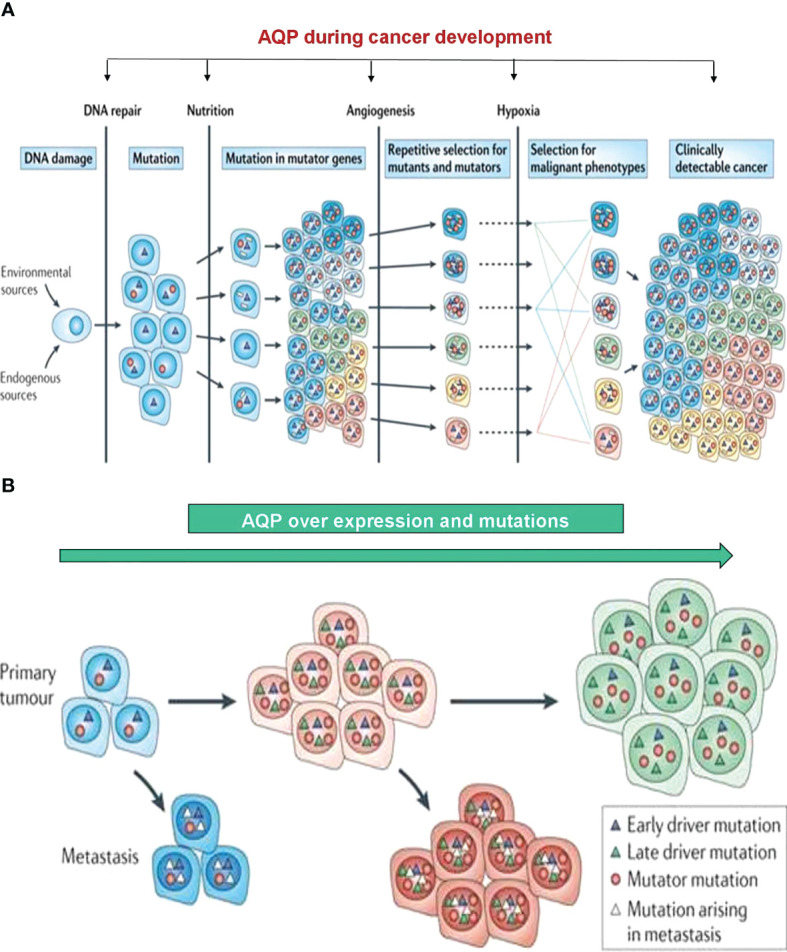
Development toward tumor growth and cancer metastasis. **(A)** Although various barriers to tumor progression exist, including DNA repair processes, accumulated mutators can promote the availability of securing nutrition, fulfilling the requirement of angiogenesis, and thus allow the tumor to increase in size. A variety of roles from AQPs during these steps are beginning to be elucidated. **(B)** AQP can play a crucial role during a series of processes, leading to various stages of accumulated DNA mutations through the activation of RAS, AKT, and MYC activations. Modified from reference (23).

Here, we briefly discuss the role of germline and sporadic mutations in the initiation and progression of human cancer and hope to provide some insight into the role of AQPs. On the basis of our initial report in 2003, we now understand that induced expression of AQPs is an early event of human carcinogenesis (23, 26, 146). Hereditary mutations among key safeguard genes underlie the presence

of genomic instability in patients with inherited cancers. The safeguard genes include DNA repair genes and mitotic checkpoint genes, which are not inactivated during carcinogenesis. As described in **Figure 4**, accumulated mutations allow tumors to increase in size and respond to various barriers for growth and metastasis. In this sense, all of the steps described in **Figure 4** are mediated through common pathways that are turned on by AQPs. Identification of “classic safe-guard genes (caretaker genes)” in sporadic cancers has been frustrating (23, 146). Of note, at least in the cases of AQPs, there have been no reports on the existence of such germline mutations. Studies of cell cycle checkpoint and DNA repair genes suggested that, in human colon cancer cell lines, these two classes of genes are seldom mutated (23, 26, 146), and genomic instability in many sporadic human cancers does not seem to be due to compromised function of caretaker genes. We are currently analyzing AQP mutations in various cancer cell lines. Our Fluorescence In Situ Hybridization (FISH) analysis examining AQP5 in resected lung cancer tissue samples did not identify a noticeable genomic amplification, but in breast cancer, we identified AQP5 genomic amplifications in more than 10% of samples (Moon et al., manuscript in press).

In summary, unlike hereditary cancers, the molecular basis of genomic instability in sporadic cancers remains unclear, and the alternative oncogene-induced DNA replication stress concept seems to be the most amenable explanation for developing sporadic cancer (23, 146). In this model, sporadic cancers may result from, among other mechanisms, the oncogene-induced collapse of DNA replication forks, leading into DNA damage and the eventual development of genomic instability. In this regard, it will be important to understand “how AQP can contribute to the development of genomic instability (23).” Clearly, on the basis of our initial reports, we now understand that the downstream pathways of AQPs, at least in the case of AQP5, might serve to turn on other pathways, including SRC-, RAS-, and AKT-mediated pathways (10, 23, 124, 148). These pathways produce DNA damage responses, which lead to the development of mutations or RNA/protein instability of key tumor suppressor genes such as p53 or p21. Perhaps over time, this will lead to accumulated mutations. Using cell lines, mouse models, and human cancer tissue samples, we have recently verified that AQP5 can turn on RAS and AKT activation, which itself carries SH3 binding activities (Kang et al., manuscript submitted; 10, 124, 148, 149).

**Figure 5:**
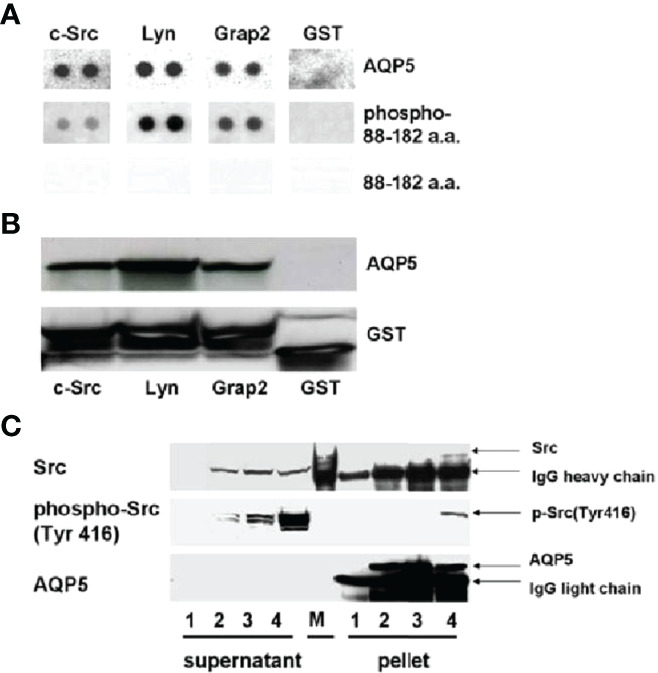
In this study, from SH3 microarray, the purified AQP5 from stable BEAS-2B cells showed binding activity to the SH3 domain of c-Src, Lyn, and Grap2 C-terminal in phosphorylation dependent manner **(A)**. GST pull-down assay confirmed a direct interaction between AQP5 and the Src family molecules **(B)**. c-Src co-immunoprecipitated with AQP5 turned out to be an activated form of c-Src, phosphorylated at Tyr416 **(C)**. Cells expressing AQP5 have more activated c-Src than the cells carrying N185D or S156A mutant **(C)**. 1, Mock; 2, N185D; 3, S156A; 4, AQP5; M, molecular weight marker. AQP5 contains SH3 binding domain, and we pointed out that the downstream pathways are very similar to the one from EGFR or other transmembrane tyrosine kinases. In cancer cells, various adapter proteins (e.g., SHC and GRB-2) can bind and subsequently turns on various downstream pathways. Adapted from reference (124).

Human AQP5 protein contains a diproline peptide sequence (RTSPVGSP) in the loop D, which carries a sequence similarity to the SH3 binding consensus site, and the report by Chae et al. (124) merits further discussion. In this study, using an SH3 microarray, the purified AQP5 showed binding activity to the SH3 domain of c-Src, Lyn, and Grap2 C-terminal in a phosphorylation-dependent manner (**Figure 5A**). GST pull-down assay confirmed a direct interaction between AQP5 and the Src family molecules (**Figure 5B**). Moreover, c-Src co-immunoprecipitated with AQP5 was found to be an activated form of c-Src phosphorylated at Tyr416 (**Figure 5C**). Cells expressing AQP5 have more activated c-Src than those carrying N185D or S156A mutant (**Figure 5C**). On the basis of this analysis and other biochemical studies, a new modeling for “how AQP5 targeting small molecules can exercise inhibitory effects on human carcinogenesis”. Such targeting may be tested by “measuring AQP binding to various adaptor molecules based on their SH3 binding activities, before and after treatments” as detailed above. Certainly, we feel that small molecules modulating such binding activity will be groups of targeted therapeutics worth future merits. Such an approach can also explain the interaction of various other targeting agents, such as EGFR targeting agents, with molecules targeting AQPs.

In fact, we have recently identified more than 50 small molecules that seem to have strong binding affinity to AQP5 in two separate sites. *In vitro* cell line assays focused on lung cancer cells are ongoing, and we plan to test promising candidate in nude mice models. In summary, we pointed out that one of the future targeting strategies should take advantage of full understanding of “how AQP can interact with these downstream pathway’s players, including various adaptor molecules, and how can we intervene these processes”. As part of expert opinion, we here provided some details of such adaptor molecules associated with AQP5.

Finally, as described in **Figures 4**, **5**, the future design of AQP-targeted therapy should be focused on various points of cancer progression. For example, we hope that AQP targeting can be used to modulate molecular pathways associated with metastasis. AQP4 has been validated as an important drug target, but no single drug has yet been approved to successfully target it. Therefore, as mentioned above, alternative routes of targeting AQP function compared with the traditional pore- blocking approach, which involves “targeting of AQPs at various points of cancer progression,” may be needed as a new AQPs targeting strategy (77, 220). Likewise, we hope that AQPs targeting can eliminate primary tumors and, particularly, rare tumor stem cells, which is one of our key targeting strategies. For this, we see enough pathway activation, leading to EMT and cancer stem cell maintenance at least by AQP5. 

## Conclusion

Here, we reviewed various reports regarding the expression patterns of AQPs in human and rodent cancer models and discussed the molecular mechanisms underlying their potential role in human carcinogenesis. Several AQPs contribute to one or more processes during each step of carcinogenesis. We have also discussed the role of AQPs in water homeostasis, particularly for AQP4 in the brain. CNS edema is frequently accompanied by brain tumors, and understanding of the role of AQP4 during CNS water transporter and lymphatic flow has made significant progress in the last decade (132, 221, 222).

So far, studies of AQP1, AQP3, and AQP5 involvements in many different cancer types are performed on the basis of numerous cell line, *in vivo* mice model, and expression profiles of these AQPs in resected cancer tissue samples. The fact that AQPs have been identified in different cancers and associated with tumor proliferation, metastasis, angiogenesis, tumor cell adhesion, and tumor-associated edema suggests that AQP family is clearly one of the key players of human carcinogenesis and now becomes an attractive therapeutic target in anticancer treatment. At this point, there is still a long way to clearly elucidate the specificity of AQPs in the pathogenesis, metabolisms, and controls of various cancers, or resistance and tolerance to anticancer therapies. However, we expect that the role of AQPs in human carcinogenesis will be continuously elucidated and understanding “why various AQPs are greatly involved in almost all types of cancers” will expand our insight regarding “how cancer develops”, “how cancer cell metastasizes”, and “how cancer stem cell develops”.

Moreover, better understanding of AQPs in cancer biology will likely result in novel anticancer therapeutics. In summary, we are cautiously anticipating that AQPs may bring in new horizons in cancer biology.

Although we did not discuss in detail in this review, the field of AQP-targeted therapeutics in cancer and possibly other metabolic diseases is still in its infancy. At least in the field of cancer therapeutics, it will be important to understand “how targets can be involved in human carcinogenesis”. For example, targeting BCR ABL in CML by Imatinib has resulted in a greater understanding of CML itself (23, 146). In this sense, questions regarding “how can AQP cause cancer development and contribute to the progression” will be expected directions of AQP research. We predict that AQP targeting will provide next-generation cancer therapeutics and that the fundamental AQP biology may well be better understood with available unique small molecules or antibodies targeting AQPs.

The hallmarks of cancer can be defined into several simple categories, and the interaction between tumor cells and the immune surveillance system plays a crucial role in tumorigenesis. Therefore, evading the immune surveillance should be an important component. Indeed, the expression of PDL1 (146), a well-known immune checkpoint molecule, in cancer surface is controlled by tyrosine kinase pathways, including Src, which we have previously demonstrated are under the control of AQP5 (124, 148, 149). In addition, several unique defense mechanisms against cellular stresses for tumor cell survival will be important. Thus, five additional hallmarks are proposed to be related to the presence of cancer stress, namely, DNA damage and DNA replication stress, oxidative stress, mitotic stress, proteotoxic stress, and metabolic stress (12, 23, 146). We further expect that AQP biology will play an important role in understanding unique interaction between tumor cells and their microenvironment.

## Author Contributions

CM was the principal investigator of the grant that supported this project. CM, DM, and SK conceived and executed the overall design of review and data collection. CM and DM wrote the paper. All authors contributed to the article and approved the submitted version.

## Funding

This study was supported, in part, by the Cancer Research Grant from Pyung-Ya Foundation (grant number PY-1; to CM) and the Translational Research Grant from HJM Foundation (grant number HJM-TR1; to CM and DM).

## Conflict of Interest

The authors declare that the research was conducted in the absence of any commercial or financial relationships that could be construed as a potential conflict of interest.

## Publisher’s Note

All claims expressed in this article are solely those of the authors and do not necessarily represent those of their affiliated organizations, or those of the publisher, the editors and the reviewers. Any product that may be evaluated in this article, or claim that may be made by its manufacturer, is not guaranteed or endorsed by the publisher.
